# Mental illness stigma among indigenous communities in Bangladesh: a cross-sectional study

**DOI:** 10.1186/s40359-023-01257-5

**Published:** 2023-07-31

**Authors:** Md. Omar Faruk, Simon Rosenbaum

**Affiliations:** 1 Department of Clinical Psychology, University of Dhaka, Dhaka, Bangladesh; 2 School of Psychiatry, Faculty of Medicine, University of New South Wales, Sydney, Australia

**Keywords:** Bangladesh, Stigma, Mental illnesses, Indigenous communities, Cross-sectional

## Abstract

**Background:**

Mental illnesses stigma is a universal and transcultural phenomenon. While mental illnesses stigma is pervasive in Bangladesh, very little research exists on stigma toward mental illnesses among indigenous communities. This study aimed to investigate the prevailing stigma and the risk factors among different indigenous communities in the Chattogram Hill Tracts (CHT) in Bangladesh.

**Methods:**

A cross-sectional survey was carried out and participants were recruited purposively from Rangamati, a South-Eastern district of Bangladesh in the CHT. Participants from various indigenous communities including Chakma, Marma, Rakhine, Tripura, and Pangkhua were recruited. The 28- item Bangla translated version of the Mental Illnesses Stigma Scale was used. Independent-samples t-test, ANOVA, and multiple regression were performed.

**Results:**

The results indicate evidence of a gender difference with females reporting more stigma than their male counterparts. Age, gender, socioeconomic status, and monthly income are associated with stigma among indigenous people. Further analyses of the subscales indicated significant differences among sociodemographic variables.

**Conclusions:**

The results provide an insight into the prevailing stigma and associate risk factors among indigenous communities. The results may help inform anti-stigma interventions targeting indigenous communities in Bangladesh.

## Introduction

More than two thirds of people with serious mental illnesses across the world do not seek treatment [1] despite the overall prevalence of mental illnesses and improved care [2, 3]. Stigma toward mental illnesses has been cited as a significant barrier to seeking mental health care [4]. Stigma generally refers to an intensely demeaning attitude, that denies people from the whole group, and excludes from full social acceptance [5]. It is also identified as a mark of shame, disgrace, and disapproval that results in discrimination or exclusion from participating in a range of areas within society [6]. Link and Phelan [7] defined stigma in light of the components that are intricately associated with the concept of stigma. They viewed stigma within the broader aspects of labeling, stereotyping, separation, status loss, and discrimination with exercising power highlighted as a major element [7]. They further explained the process by which stigma takes place. At the outset people differentiate and label others which is then used to label people to undesirable characteristics by dominant cultural beliefs. Separating ‘us’ from ‘them’ occurs when labeled people are placed in distinct categories to accomplish the diversion. In the subsequent phase, labeled people experience status loss and discrimination leading to unequal outcomes. In the end, the restricted access to social, economic, and political power emerged from stigmatization perpetuates the process of differentness, continued construction of stereotypes, categorization of labeled people, and the full-flagged execution of disapproval, rejection, exclusion, and discrimination [7].

Multiple types of stigmas related to mental illnesses were identified including self-stigma, public stigma, professional stigma, and institutional stigma. When an individual demonstrates a negative attitude toward his/her own mental illnesses, it is called self-stigma and often used interchangeably with internalized stigma [8, 9]. This results in another form of stigma, label avoidance, in which the individual interprets mental illnesses as ‘crazy’, therefore, refrains from seeking help [10]. Negative attitude held by general people demonstrated toward those with mental illnesses is characterized as public stigma [8, 9]. Professional stigma refers to the stigmatized attitudes toward patients with respect to the faulty understanding of the causes and symptoms of mental illnesses. It also occurs when professionals experience stigma from general people or health care professionals due to the work nature and association with the individuals already classified as having mental illnesses [8]. Finally, institutional stigma characterizes the policies or cultures responsible for fostering negative attitudes and beliefs toward people with mental illnesses [11–14]. Each type of stigma represents a myriad of consequences in various aspects of life. For example, stigma impairs the experience of self-efficacy, self-esteem, employment, relationship, social status, altered behavioral presentation, treatment-seeking behavior, and physiological complaints (e.g., obesity, back pain, and sexual dysfunctions) [3, 8]. In addition, mental illnesses stigma disrupts care and life opportunities (e.g., work and housing) [6]. Subject to stigma lowers the quality of life [15] and can increase risk of poorer health outcomes including premature death [16, 17]. Evidence also suggests that people with mental illnesses are more likely to be objected from making choice or opinions unlike persons with physical illnesses [18]. Besides, stigma toward mental illnesses can include a perception whereby mental illnesses is incorrectly associated with substance misuse, prostitution, and criminality [19]. Previous research has shown that people with psychotic disorders were perceived to have control over their conditions and responsibility in causing those [20]. Withholding help to some minority groups due to stigma is also evident [21]. People with mental illnesses experience difficulty in coping with the illnesses and stigma or discrimination derived from a lack of societal understanding of mental health disorders [21, 22]. The interplay of the nature of illnesses and the stigma, as well as the resulting discrimination, challenges people with mental illnesses with the opportunity in ensuring education and employment, safe housing, health care, and social connection [21]. Stigma and its consequences have been described by people with mental illnesses as being worse than the condition itself [23]. This widespread stigma in turn can lead to ‘secondary illnesses’ after being labeled with mental illnesses causing subsequent untoward consequences in various aspects of life [6].

Although stigma is a transcultural phenomenon, the origin and risk factors are not universal and vary across cultures in which multiple risk factors work synergistically [24]. For example, Ng [25] showed that stigma toward mental illnesses is prevalent in Chinese, Indian, Japanese, South-East Asian, and Islamic cultures. Evidence showed that stigma toward mental illnesses was present among American Indians and Alaska Native people [26]. In Australia, stigma is also found to be present among indigenous people [27]. Differences in stigma have also been reported among various cultural groups such as American or American Indians, Asian, African, Latino, Middle Eastern, and European descent [28]. However, evidence suggests that the degree of stigma toward mental illnesses varies across cultures. For example, stigma seems to be less prevalent in Asian and African countries and Islamic societies compared to the Western world [21]. Some argued that personal, social, familial factors, and even the nature of the illnesses can facilitate stigma [29]. Others have highlighted the role of low education and awareness, perception, and the interaction of the nature and complications of mental illnesses [24].

For millennia, mental illnesses were considered a sin as well as a crime in majority of the societies and cultures for which people with mental illnesses were chained and imprisoned, and tortured. The widespread abuse toward people with mental illnesses continued during the Middle ages, Enlightenment period, and even in the modern period when people with mental illnesses were murdered or sterilized during the Nazi reign in Germany [30]. Stigma toward mental illnesses has remained a major public health concern worldwide despite the strengthening of mental health care [31]. While some progress has been made in specific contexts across the world to reduce mental illnesses stigma, the study of stigma toward mental illnesses in Bangladesh has remained largely under-recognized, especially among indigenous communities.

In Bangladesh, it was reported that widespread stigma has contributed to lower access to mental health care [32]. The National Mental Health Survey found that people with mental health problems avoided seeking mental health treatment due to stigma (e.g., labeling with derogatory terms such as people with mental illnesses are mad) [33]. Mental illnesses in Bangladesh are generally seen as a consequence of possession of evil spirits without recognition of biological or psychological markers as causes of mental illnesses [33]. These superstitious beliefs about the causation of mental illnesses have far-reaching implications in terms of help-seeking behaviors. For example, seeking help from traditional healers is common in Bangladesh when it comes to treating mental illnesses [33] irrespective of geographical locations and ethnicities including indigenous people. Evidence suggests that indigenous people in Bangladesh also tend to have superstitious beliefs about the cause and perpetuation of mental illnesses [34]. However, little study exists to report the prevailing stigma and the risk factors responsible for the perpetuation of stigma among indigenous people in Bangladesh, especially, in the Chattogram Hill Tracts (CHT) regions.

Bangladesh is home to 49 indigenous communities. Most live in the CHT- the Southeastern part of the country comprising three hill tracts districts such as Rangamati, Bandarban, and Khagrachari. The population in the CHT is estimated at 1.6 million comprising approximately 2% of the total Bangladesh population [34]. The remaining indigenous communities live in the northern part of Bangladesh. Each indigenous community has distinct customs and values. Various beliefs and customs are present in relation to physical and mental health problems among indigenous communities. For example, supernatural power is believed to influence birth, death, and all activities in life in the Marma community while sacrificial animals (e.g., goats, chickens, and ducks) are thought to prevent bad spirits causing physical and mental illnesses in the Chakma community [34]. Mental health literacy including knowledge of mental health care is largely absent among indigenous communities [34]. Therefore, mental health symptoms are often perceived and reported as somatic problems that are treated by embracing rituals to satisfy the spirits believed to be causing such problems [34]. While there is evidence suggesting that stigma toward mental illnesses is common in indigenous communities in other countries such as Australia [35], little is known about stigma toward mental illnesses among different indigenous communities in Bangladesh. Limited scholarly work has reported the presence of mental illnesses stigma in Chakma and Marma communities [28], however, mental illness stigma among other indigenous communities has remained insufficiently studied. Therefore, the present study aimed to investigate the prevailing stigma among different indigenous communities in Bangladesh and the factors associated with stigma toward mental illnesses. The study attempted to understand the difference in stigma in terms of gender, prior knowledge about mental health, the presence of a family member with mental illnesses, and the treatment seeking behavior. Furthermore, the study also attempted to understand the difference in terms of anxiety, relationship disruption, hygiene, treatability, personal efficiency, and recovery- the seven subscales of the scale used in the present study. The study is expected to contribute to the pan-cultural understanding of stigma toward mental illnesses by investigating stigma among indigenous communities in Bangladesh.

## Methods

### Study population

**Fig. 1:**
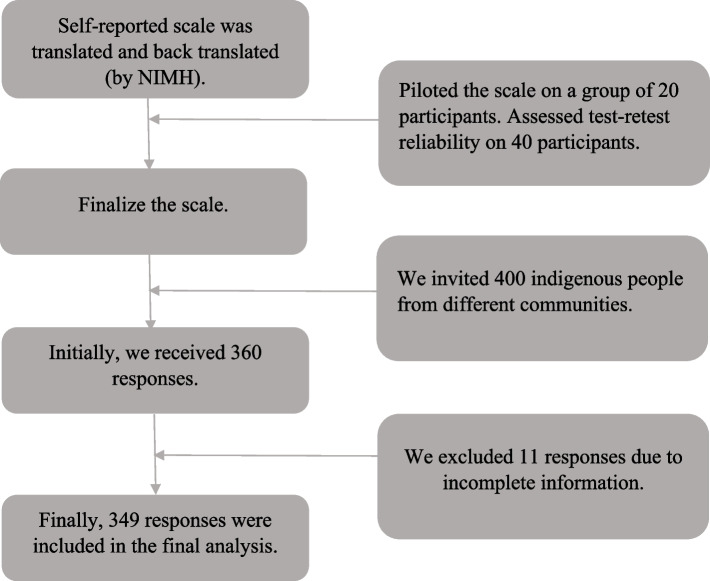
Flowchart of collecting responses

A total of 349 participants from different indigenous communities were purposively recruited from the Rangamati division and adjacent villages. Representation of the major indigenous communities, feasibility of recruiting and retaining participants within the study period, and the geographic distribution in Rangamati were taken into consideration while recruiting participants. Approaching participants living in the remote hilly areas presented practical constraints. With these issues in mind data from 360 participants were collected. The majority of the Chakma people, the predominant community in the CHT live in Rangamati while others live in other divisions in the CHT. The flowchart of collecting data from the participants is presented in Fig. 1.

### Measures

The following measures were used in the study.

### Sociodemographic measures

The sociodemographic measures included age, gender, ethnicity, religion, educational level, relationship status, income, occupation, socioeconomic status (SES), presence of persons with mental illnesses in the family, knowledge about mental health, and history of treatment for mental health (Yes/No). The history of mental illnesses was assessed using vignette of common mental health disorders along with schizophrenia and bipolar mood disorders. SES was assessed with a self-reported question pertaining to the financial sufficiency such as “how would you classify your socioeconomic status for the household food consumption over the last 12 months?” [36, 37]. The possible responses were a) insufficient funds for the whole year; b) insufficient funds some of the time; c) neither a surplus nor a deficit; d) sufficient funds most of the time. Based on the responses, SES was categorized into four levels (lower SES, lower-middle SES, middle SES, and higher SES). Psychometric tools with a view to assessing mental health awareness are scarce in Bangladesh, therefore, knowledge about mental health was assessed using a single item question (*I have knowledge about mental health*) with a *Yes* and *No* response format.

### The Bangla translated version of the mental illness stigma scale

The Bangla translated version of the Day’s Stigma Scale [38] was used in the study. The Bangla version of the scale was used in the National Mental Health Survey in 2019. The scale was pretested by the National Institute of Mental Health and Hospital (NIMH) on a sample of 300 participants prior to the use in final data collection. No published data on the psychometric properties of the scale is available. However, the present study recruited 40 participants (excluding the total sample size in the study) from diverse indigenous communities (e.g., Chakma, Marma, Rakhine, Tripura, and Pangkhua) in order to assess test–retest reliability with a gap of two weeks prior to the administration of the scale. The test–retest reliability for the Bangla version of the scale for this study was found to be, r = 0.98 (*p* < 0.01) suggesting an excellent value [39]. The higher test–retest coefficient may be attributed to the stability of the construct under consideration as constructs such as attitudes, beliefs, or personality traits are generally considered stable over time. Furthermore, the test–retest assessment was conducted with homogenous participants with similar geographical location and educational attainment (the participants were university students). Homogeneity may have minimized the potential for external factors to influence responses and enhanced the consistency of participant responses over time. The Cronbach’s alpha on a sample of 349 participants was α = 0.74. indicating an acceptable value [40]. The scale assesses attitude toward persons with mental illnesses in terms of seven factors: interpersonal anxiety, relationship disruption, poor hygiene, visibility, treatability, professional efficacy, and recovery. The seven-point Likert-type scale ranges from 1 (completely disagree) to 7 (completely agree). Item no. 8, 9, 11, 13, and 20 contain reverse scoring. A higher score represents higher mental illnesses stigma and vice versa.

### Data collection procedure

Two undergraduate psychology students (research assistants) collected the data. They were trained by the first author prior to commencing data collection. The training contents included the types of mental illnesses, understanding of stigma, and cognitive interviewing through role play. The training involved cognitive interviewing to understand the ways in which participants mentally process and respond to survey questionnaires [41]. In addition, the purpose of cognitive interviewing was to ensure that true meaning of the questionnaires, as intended by the researchers, was conveyed to participants. The research assistants were members of Chakma and Marma communities, the largest and second-largest indigenous communities in the CHT, respectively. Besides Chakma, Marma and Bangla language, the research assistants spoke other languages (i.e., Rakhine, Pangkhua, and Tripura). Participants were also able to speak Bangla dialect- the national language in Bangladesh besides their distinct dialects. We chose to select research assistants from indigenous communities due to their cultural competence, knowledge, values, norms, insights, and language proficiency allowing for more accurate and culturally appropriate interactions with participants. Recruiting research assistants from the same community may help ensure participants feel comfortable sharing experiences, perspectives, and sensitive information to those who share the same background and can relate to their experiences. Furthermore, involving research assistants from the community helps foster community engagement and empowerment. It demonstrates a commitment to community involvement and recognizes the importance of local voices and perspectives in research.

The scale was originally developed by Days et al. [38]. The Bangla version was prepared by the National Institute of Mental Health and Hospital (NIMH) in Dhaka following standard translation and back-translation procedures. The scale was pretested with a group of 20 indigenous people from diverse indigenous communities (Chakma, Marma, Rakhine, and Tripura) with a view to checking the understanding of each item prior to the administration on indigenous people.

Several indigenous communities live in Rangamati such as Chakma, Marma, Tanchangya, Tripura, Pangkhua, Lushai, Khiang, Murang, Rakhine, Chak, Bawm, and Khumi. However, the largest indigenous communities (Chakma, Marma, Tripura, Rakhine, and Pangkhua) in Rangamati were selected for data collection. Participants were purposefully selected across different geographical locations including the remote areas in Rangamati based on some characteristics such as residents in the CHT, member of indigenous communities, and ≥ 18 years of age. Purposeful sampling strategy was deemed feasible due to the hard-to-reach nature of the participants, especially those (e.g., Pangkhua and Rakhine) live in the remote hill tracts regions. Research assistants made home visits and collected the data upon meeting the characteristics and obtaining verbal consent using the native language. Participants were given a verbal and written consent form. The potential benefits, risks, and the right to withdraw from providing data was explicitly mentioned in the consent orientation and in the written form. We used clear and simple language in consent forms, instructions, and questionnaires avoiding jargon and technical terms for participants with little or no literacy. Research assistants were asked to present oral information in a concise and straightforward manner and record the responses. Additionally, they were asked to focus on clear communication and sensitivity to the needs and challenges faced by participants with little or no literacy. Assistance was offered to participants with little formal education who had difficulty reading or comprehending the scale. This involved reading the items aloud, providing explanations when needed, or offering additional support to ensure participants understood the rating options. Being the representatives of some of the communities, the research assistants were able to act as intermediaries helping to bridge the communication gap and facilitate data collection. A thumb mark was used to indicate consent for participants with no literacy. On average, it took 35 min to complete the survey. Participation in the study was voluntary, therefore, no monetary compensation was provided. Data were collected between August and September 2021 when the countrywide lockdown to reduce the spread of COVID-19 was lifted.

### Data analysis

SPSS 25 was used to analyze data. Demographic variables were analyzed by descriptive statistics (frequencies, percentages, and means). Independent-samples *t*-test was used to investigate gender differences, people with and without knowledge about mental health, and people with and without a family member with mental illnesses. Multiple regression analysis was performed to identify variables that are associated with stigma toward mental illnesses. Findings of multiple regression were interpreted with 95% confidence intervals (at *p* < 0.05).

### Ethical considerations

The study was conducted in accordance with the guidelines outlined in the Helsinki Declaration. The study was approved by the ethical review committee at the Department of Clinical Psychology, University of Dhaka (Project ID: IR210601). Participants were given a list of available mental health services in case they experienced distress during or after data collection.

## Results

Data from 360 participants were collected, with 11 participants excluded from the analysis due to incomplete responses. Therefore, the final sample included 349 people aged between 18 and 80 years (Mean 36.9; SD 15.2) (34% female; 66% male). Analysis of sociodemographic variables suggested that about 17% of all participants had some knowledge about mental health, 3% of participants had a family member with mental illnesses, and 4% of the total participants had sought mental health treatment at some time in their lives. The majority of participants were Buddhist (95.7%) as well as married (55.0%). The remaining sociodemographic properties are presented in Table 1.

**Table 1:** Demographic properties of participants

Participants Characteristics	*N* = 349 (%)
Ethnicity	
Chakma	165 (47.3)
Marma	102 (29.2)
Rakhine	31 (8.9)
Tripura	28 (8.0)
Pangkhua	23 (6.6)
Gender	
Male	231 (66.2)
Female	118 (33.8)
Socioeconomic Status (SES)	
Lower SES	81 (23.2)
Lower-middle SES	125 (35.8)
Middle SES	131 (37.5)
Higher SES	12 (3.4)
Occupation	
Student	88 (25.2)
Service Holder	63 (18.1)
Businessperson	85 (24.4)
Housewife	69 (19.8)
Unemployed	44 (12.6)
Marital Status	
Unmarried	134 (38.4)
Married	192 (55.0)
Widow/widower	23 (6.6)
Religion	
Buddhist	334 (95.7)
Christian	15 (4.3)
Literacy	
Up to primary	42 (12.0)
Primary to secondary	44 (12.6)
SSC*	55 (15.8)
HSC**	81 (23.2)
Hons’	66 (18.9)
Master’s and above	36 (10.3)
Illiterate	25 (7.2)
Knowledge about mental health	
Yes	60 (17.2)
No	289 (82.8)
Presence of mental illness among family members	
Yes	9 (2.6)
No	340 (97.4)
Treatment sought for Mental Illness	
Yes	15 (4.3)
No	334 (95.7)

^*^SSC is the abbreviation of Secondary School Certificate

^**^HSC is the abbreviation of Higher Secondary Certificate

An independent-samples *t*-test was conducted to explore gender differences in stigma in relation to the total score. A statistically significant difference between males (M = 128.12, SD = 15.614) and females (M = 134.26, SD = 14.260) was found *t*(347) = -3.580, *p* < 0.05). No significant differences in terms of the level of stigma was found between participants with (M = 130.60, SD = 14.468) and without knowledge (M = 130.11, SD = 15.640); at *p* > 0.05 about mental health. Similarly, no significant differences among participants with (M = 125.33, SD = 18.621) or without a family member with mental illnesses (M = 130.32, SD = 15.344); at *p* > 0.05, and whether treatment for mental illnesses was sought (M = 123.73, SD = 15.957) or not (M = 130.49, SD = 15.363); *p* > 0.05 were found.

Mental illnesses stigma scores were reported in accordance with the subscales—anxiety, relationship disruption, hygiene, visibility, treatability, professional efficiency, and recovery. Preliminary analyses were conducted to ensure no violation of the assumptions of normality and multicollinearity (Tolerance > 0.10 and VIF < 10). We applied Bonferroni correction for adjusting the significance level for multiple comparisons to control the overall Type I error rate.

The results showed that there was significant mean difference of multiple subscale scores for mental illnesses stigma [Treatability: *F* (2,346) = 6.01, *p* < 0.01; Relationship disruption: *F* (2,346) = 12.75, *p* < 0.01; Hygiene: *F* (2,346) = 9.93, *p* < 0.01; Anxiety: *F* (2,346) = 25.65, *p* < 0.01; Visibility: *F* (2,346) = 5.00, *p* < 0.01; Professional efficiency: *F* (2, 346) = 3.39, *p* < 0.05] among the age groups. The results also showed that for relationship disruption, the mean difference of participants aged > 25 years was significantly different than participants aged > 40 years for relationship disruption while participants aged between 25 and 40 also had significant mean difference from participants aged > 40 years. Additionally, participants aged > 25 years had significant mean difference with participants aged between 25 and 40 as well as > 40 years for Hygiene subscale. In case of anxiety subscale, participants irrespective of age range (i.e., < 25, 25–40, and > 40) had significant mean difference with each other. Finally, for visibility subscale participants < 25 years of age had significant mean difference with participants aged > 40 years.

In case of relationship disruption, participants with no formal education and who had educational attainment up to secondary level had significant mean difference with participants having hons’ level of education. For anxiety subscale, there was a significant mean difference between participants education up to HSC and Hons’ and participants with no formal education. For recovery subscale, participants having MS degree and above had significant mean difference with participants having no formal education. Finally, participants with no formal education had significant mean difference with participants having educational attainment up to secondary, hons’, and MS & above for professional efficiency.

Participants belonging to lower SES had significant mean difference with participants of lower-middle SES while lower-middle SES group had mean difference with middle SES for treatability subscale. Middle SES groups had significant mean difference with lower SES in case of relationship disruption. For hygiene subscale, lower SES groups and lower-middle SES had mean difference with middle SES groups. Middle SES groups had significant mean difference with lower SES groups for anxiety subscale. Lower SES groups had significant mean difference with lower-middle groups both for recovery and professional efficiency subscales.

For treatability subscale, the analysis showed that there is a significant mean difference between Pangkhuas and Rakhines. For visibility subscale, Chakma participants showed significant mean difference with Marmas and Rakhines. Besides, there was a significant difference between Rakhines and Pangkhuas. The Tukey HSD post hoc comparison showed that there was a significant mean difference between Rakhine and Pangkhua participants for recovery subscale.

Age, gender, socioeconomic status, and monthly income were associated with stigma toward mental illnesses (Tables 2 and 3). The regression model was significant *F*(13, 347) = 5.614, *p* < 0.05, and in combination, these demographic variables accounted for 17.9% of the variability (R Square) in stigma. Multicollinearity, when multiple independent variables are correlated, was checked when performing the regression analysis. Because correlation between independent variables may affect the accurate estimation of the regression model, its results and interpretations [42]. The possible remedy for multicollinearity involves the removal of highly correlated variables from the analysis, transforming highly correlated variables into a single composite score, or performing modelling approaches such as Principal Component Analysis (PCA) to decompose data into a number of independent variables [42]. While some researchers prefer variable pruning by means of PCA, others transform the correlated variables into a single index. However, both of these approaches have been criticized due to the loss of interpretability of the individual contribution of the variables to the dependent variable [43, 44]. We investigated the probability of multicollinearity through the correlation matrix of the independent variables. The correlation coefficients revealed a higher correlation between occupation and gender (0.54, *p* < 0.01). Literature suggests that removal of one variable can be considered when the correlation between independent variables is around 0.7 [45]. Based on the correlation matrix, the independent variables were retained in the regression model. It is important to note that income is often considered a significant component of SES and can have a strong impact on the overall socioeconomic standing. Changes in income can affect SES, and variations in SES can reflect differences in income levels among individuals or groups. Therefore, monthly income and SES were considered as predictor variables in regression analysis despite their interchangeable nature.

**Table 2:** ANOVA for subscales

Variables	Treatability		RD		Hygiene		Anxiety		Visibility		Recovery		PE	
	M (SD)	F (df)	M (SD)	F (df)	M (SD)	F (df)	M (SD)	F (df)	M (SD)	F (df)	M (SD)	F (df)	M (SD)	F (df)
Age														
< 25	16.10 (3.9)	6.16** (2,348)	27.08 (6.8)	12.75** (2,348)	20.46 (5.1)	9.93** (2,348)	31.08 (8.5)	25.66** (2,348)	16.92 (4.4)	5.00** (2,348)	5.05 (3.1)	0.91 (2,348)	11.15 (3.0)	3.39* (2,348)
25–40	15.48 (4.2)		28.43 (6.1)		21.98 (4.2)		34.46 (6.6)		16.16 (4.1)		5.45 (3.4)		11.96 (2.5)	
> 40	16.06 (3.1)		30.97 (4.9)		22.98 (3.3)		38.02 (6.6)		15.27 (3.3)		4.95 (2.6)		11.90 (1.9)	
Ethnicity														
Chakma	11.16 (2.9)	3.82** (4,348)	29.67 (6.1)	1.78 (4,348)	22.04 (4.6)	1.07 (4,348)	35.45 (7.7)	.82 (4,348)	15.22 (3.9)	6.43** (4,348)	4.95 (2.9)	2.79* (4,348)	11.81 (2.3)	.24 (4,348)
Marma	11.84 (3.2)		28.26 (6.1)		21.73 (3.7)		34.29 (7.9)		16.61 (3.9)		5.47 (3.2)		11.63 (2.6)	
Rakhine	10.06 (3.2)		27.16 (6.2)		23.10 (3.9)		33.23 (7.2)		18.45 (3.2)		4.13 (2.9)		11.81 (2.9)	
Tripura	11.14 (3.1)		29.29 (5.9)		20.93 (4.9)		35.71 (6.9)		16.86 (4.1)		5.11 (3.3)		11.86 (2.8)	
Pangkhua	13.00 (3.6)		30.04 (5.2)		22.09 (3.5)		35.13 (7.2)		14.87 (3.0)		6.70 (3.1)		11.35 (2.4)	
Gender														
Male	11.66 (3.4)	5.29* (1, 348)	28.29 (6.1)	1041** (1, 348)	21.37 (4.3)	13.66** (1, 348)	33.92 (7.8)	11.87** (1, 348)	16.16 (4.1)	0.89 (1, 348)	5.02 (2.9)	1.32 (1, 348)	11.69 (2.6)	.18 (1, 348)
Female	10.85 (2.5)		30.48 (5.8)		23.11 (3.8)		36.86 (6.9)		15.75 (3.7)		5.42 (3.4)		11.80 (2.3)	
Occupation														
Service	12.22 (3.9)	1.64 (4,348)	28.78 (5.7)	5.73** (4,348)	21.56 (4.0)	5.58** (4,348)	35.05 (6.2)	9.63** (4,348)	16.16 (3.5)	2.90* (4,348)	4.86 (2.8)	1.78 (4,348)	12.06 (2.4)	2.84* (4,348)
Business	11.14 (2.7)		30.24 (5.1)		22.38 (3.6)		36.73 (6.8)		15.78 (3.7)		5.13 (2.9)		12.28 (1.8)	
Unemployed	11.61 (3.4)		21.91 (6.1)		21.66 (3.8)		33.25 (8.1)		15.66 (4.1)		5.70 (3.2)		11.02 (2.8)	
Student	11.05 (3.2)		27.05 (7.1)		20.65 (5.2)		31.52 (8.3)		17.09 (4.5)		4.65 (3.1)		11.35 (3.0)	
Housewife	11.20 (2.6)		31.03 (5.3)		23.67 (3.5)		37.94 (6.8)		15.07 (3.4)		5.77 (3.4)		11.67 (1.9)	
Socioeconomic Status (SES)														
Lower SES	11.99 (2.9)	9.25** (3, 348)	31.16 (4.8)	5.94** (3, 348)	23.10 (3.3)	13.07** (3, 348)	37.35 (7.4)	5.704** (3, 348)	15.33 (3.9)	1.12 (3, 348)	6.05 (2.8)	5.44** (3, 348)	11.20 (2.3)	2.91* (3, 348)
Lower-middle SES	10.25 (2.2)		29.14 (6.2)		23.06 (3.9)		35.03 (8.0)		16.15 (3.8)		4.36 (2.8)		12.14 (2.1)	
Middle SES	11.99 (3.70)		27.61 (6.4)		20.26 (4.6)		33.11 (7.2)		16.29 (3.9)		5.33 (3.3)		11.60 (2.8)	
Higher SES	12.50 (3.6)		29.00 (4.5)		21.96 (3.3)		37.00 (4.5)		16.42 (4.3)		5.58 (3.3)		1.73 (2.5)	
Literacy														
Up to primary	11.57 (2.7)	1.91 (6, 348)	28.12 (6.6)	4.3** (6, 348)	22.90 (3.6)	2.7* (6, 348)	34.48 (9.9)	3.5** (6, 348)	16.10 (4.4)	1.33 (6, 348)	6.19 (3.4)	2.85* (6, 348)	11.67 (2.1)	3.3** (6, 348)
Up to secondary	11.18 (2.9)		30.73 (4.4)		22.98 (4.0)		36.09 (6.9)		15.57 (4.3)		5.11 (2.9)		11.95 (1.7)	
SSC	11.18 (2.8)		29.91 (5.2)		22.15 (4.1)		35.49 (6.5)		16.24 (2.8)		4.69 (2.6)		11.65 (2.4)	
HSC	11.02 (3.0)		29.23 (5.8)		21.36 (4.1)		34.75 (7.9)		16.38 (3.9)		4.99 (3.2)		11.60 (2.7)	
Hons’	10.95 (3.4)		26.67 (6.9)		20.79 (5.2)		32.41 (8.2)		16.67 (4.3)		4.88 (3.4)		11.95 (2.9)	
Master’s and above	12.33 (4.1)		28.17 (6.8)		21.61 (3.9)		34.50 (5.2)		14.94 (3.9)		4.42 (2.7)		12.69 (1.7)	
Illiterate	12.80 (2.6)		32.48 (4.7)		23.68 (2.5)		40.04 (4.7)		14.92 (3.1)		6.88 (2.4)		10.00 (2.5)	
Religion														
Buddhist	11.43 (3.1)	1.55 (1, 348)	28.98 (6.1)	0.58 (1, 348)	21.90 (4.3)	1.35 (1, 348)	34.95 (7.6)	.19 (1, 348)	16.03 (3.9)	0.03 (1, 348)	5.16 (3.1)	.01 (1, 348)	11.74 (2.5)	.28 (1, 348)
Christian	10.40 (2.7)		30.20 (5.4)		23.20 (2.9)		34.07 (8.3)		15.87 (3.8)		5.13 (3.1)		11.40 (1.8)	
Knowledge about mental health														
Yes	10.87 (2.8)	1.98 (1, 348)	28.52 (6.8)	.52 (1, 348)	22.95 (3.8)	4.01* (1, 348)	34.83 (7.8)	.01 (1, 348)	16.98 (4.1)	4.38* (1, 348)	4.05 (3.1)	9.54** (1, 348)	12.40 (2.2)	5.44* (1, 348)
No	11.49 (3.2)		29.14 (5.9)		21.75 (4.3)		34.93 (7.6)		15.82 (3.9)		5.39 (3.0)		11.59 (2.5)	
Presence of mental illness among family members														
Yes	10.67 (4.4)	.48 (1, 348)	29.67 (6.5)	.10 (1, 348)	18.56 (5.5)	6.03* (1, 348)	33.44 (7.7)	.34 (1, 348)	16.11 (5.2)	.01 (1, 348)	5.22 (3.4)	.004 (1, 348)	11.67 (2.6)	.006 (1, 348)
No	11.40 (3.1)		29.01 (6.1)		22.05 (4.2)		34.95 (7.6)		16.02 (3.9)		5.16 (3.1)		11.73 (2.5)	
Treatment sought for Mental Health Illness														
Yes	10.40 (2.4)	1.55 (1, 348)	29.00 (5.1)	.00 (1, 348)	22.03 (4.2)	2.31 (1, 348)	32.47 (5.8)	1.61 (1, 348)	15.67 (4.7)	0.13 (1, 348)	5.33 (3.4)	.05 (1, 348)	10.53 (2.5)	3.70 (1, 348)
No	11.43 (3.2)		29.03 (6.1)		20.33 (5.8)		35.02 (7.7)		16.04 (3.9)		5.15 (3.1)		11.78 (2.5)	

*df* degrees of freedom, *SD* Standard deviation

^*^p-value < 0.05

^**^p-value < 0.01, *RD* Relationship disruption, *PE* Professional efficiency, *M* Mean

**Table 3:** Regression coefficients of demographic variables on stigma toward mental illness with R^2^ = 17.9

Variables	B	SE	*t*	*p*	95% CI	
					Lower Bound	Upper Bound
Ethnicity	0.669	0.645	1.037	0.301	-0.600	1.938
Age	0.239	0.055	4.343	0.001	0.131	0.348
Gender	8.354	2.006	4.164	0.001	4.407	12.300
Occupation	-0.939	0.791	-1.186	0.236	-2.495	0.618
Marital status	-0.302	1.085	-0.278	0.781	-2.437	1.833
Treatment sought for mental illness	-5.781	3.853	-1.500	0.135	-13.361	1.799
Number of family members	0.043	0.512	0.084	0.933	-0.964	1.049
SES	-3.775	1.059	-3.566	0.001	-5.858	-1.693
Presence of MH patients in the family	-6.609	5.046	-1.310	0.191	-16.535	3.317
Monthly income	0.00	0.000	1.958	0.040	0.000	0.000
Knowledge about MH	2.189	2.062	1.062	0.289	-1.867	6.245
Educational	0.024	0.483	0.050	0.960	-0.925	0.974
Religion	-0.114	3.843	-0.030	0.976	-7.674	7.446

Multiple regression was also conducted in terms of seven subscales with demographic variables such as ethnicity, gender, age, SES, occupation, number of family members, marital status, religion, literacy, knowledge about mental health, presence of mental illnesses among family members, and treatment sought for mental illnesses as independent predictors. For treatability subscale the regression model was found to be significant [*F*(13, 347) = 2.906, *p* < 0.05)] with all independent variables accounting for 10.2% of the variability in mental illness stigma. In addition, the number of family members and monthly income were found to be associated with treatability. The demographic variables accounted for 13.8% of the variability for the subscale relationship disruption [regression model: *F*(13, 347) = 4.120, *p* < 0.05] while only age, gender, and SES were found to be significantly associated with relationship disruption. In case of the subscale hygiene, the demographical variables altogether accounted for 18.8% of the variability [regression model: *F*(13, 347) = 5.939, *p* < 0.05] with age, gender, SES, and prior knowledge about mental health significantly associated with hygiene. For anxiety subscale, the independent variables accounted for 18.6% of the variability [regression model: *F*(13, 347) = 5.884, *p* < 0.05] with age, gender, and SES as significant associated factors. Professional efficiency accounted for 8.1% of the variability [regression model: *F*(13, 347) = 2.259, *p* < 0.05] considering all demographic variables as independent predictors while marital status, prior knowledge about mental health and illness, and treatment sought for mental illnesses were found to be significantly associated with professional efficiency. In the case of visibility and recovery subscales, the regression models were found to be statistically not significant.

## Discussion

This study is among the first to report data on stigma toward people with mental illnesses among five indigenous communities in the CHT of Bangladesh. The study recruited 349 participants by means of purposive sampling. The Bangla translated version of the Day’s Stigma Scale was used to collect data. We found evidence of a gender difference and socioeconomic variables (gender, age, SES, and income) are risk factors of stigma toward mental illnesses among indigenous people.

We found evidence of a gender difference in the experience of stigma with females reporting more stigma toward mental illnesses than males. The results are in line with previous studies in which females living in the rural areas in America and Western societies held more stigmatizing or unfavorable attitudes toward mental illnesses compared to their male counterparts [46, 47]. In contrast, numerous studies have also found that females in general tend to have less stigma toward mental illnesses than males [48–52] including having more humanitarian attitude toward people with mental illnesses [53]. However, it is of note that those studies were carried out among non-indigenous medical students having prior personal contact with persons with mental illnesses [50] or primary school students [49]. Little is known about the gender differences in stigma among indigenous communities across the world including in Bangladesh. The reason why females reported more stigma than males may be attributed to the varying nature of coping behaviors. Evidence showed that females tend to use informal support such as family and friends [54]. This informal help-seeking behaviors may prevent females from seeking proper mental health care. Our findings may inform anti-stigma campaigns in the CHT and suggest that gender-specific outreach programs should be considered. Further qualitative research is needed to explore the gender differences in stigma and the social as well as other cultural factors that determine the higher degree of stigma in females than males belonging to indigenous communities.

Age was found to be associated with stigma toward mental illnesses converging with the existing body of knowledge on stigma [47, 55]. Previous research has showed that stigma toward mental illnesses change with age. For example, stigma was found to decrease among school children with increasing age [49, 56]. However, not all studies demonstrated the interrelationship of increasing age and lower stigma of mental illnesses suggesting an inconclusive support for age and stigma toward mental illnesses. In their study conducted in Sweden with data from 1974 to 2014 Mirnezami et al. [2] found that participants less than 20 years of age showed less stigma than older age groups. This implies that culture may also dictate the degree of stigma among various age groups suggesting that the notion of mental illnesses stigma reduces with increasing age is an overgeneralization. There is a reason to believe that the association between age and stigma is overgeneralized in the face of limited scholarly work carried out among indigenous communities across the world. The investigation of stigma toward mental illnesses in relation to age among indigenous communities is scarce worldwide. However, our study provides evidence that age and mental illnesses stigma are positively correlated in line with findings of previous studies [47, 55, 56].

Results of the present study also suggest that socioeconomic status was associated with stigma toward mental illnesses in line with existing research [57–59]. Evidence suggests that higher SES was associated with greater mental illnesses stigma [58, 59]. In contrast, other studies have showed that people with lower SES express more mental illnesses stigma in England and other parts of the world [60]. Studies looking at the association of SES and stigma among indigenous communities is scant worldwide including in Bangladesh. Hence, qualitative studies are required to understand how SES moderates the association between SES and stigma among indigenous people in Bangladesh.

Income was found to be another associated factor of stigma toward mental illnesses contributing to the existing evidence in which higher income is associated with greater levels of mental illnesses stigma [57]. Those with higher incomes may perceive themselves as more socially privileged and may stigmatize individuals with mental illness as a way to maintain their social status or distance themselves from perceived vulnerability. This can lead to the reinforcement of negative stereotypes and discrimination against individuals with mental health issues. It is important to note that the relationship between income and mental illness stigma is not deterministic and can vary across different contexts and populations. Further research is needed to better understand the complex interplay between socioeconomic factors and mental illnesses stigma, especially among indigenous people.

Although it has been shown that knowledge about mental health and contact with people experiencing mental illnesses were associated with lower levels of stigma [61, 62], these factors failed to demonstrate any association with stigma toward mental illnesses among indigenous people in the current study. Indigenous people in the CHT may have different understanding about the origin and perpetuation of mental illnesses. For example, indigenous communities in the CHT (e.g., Chakma and Marma) subscribe to supernational entities as the causation of mental illnesses, therefore, offer sacrificial animals to calm the entities [34]. Lack of mental health literacy and access to mental health care in CHT may also have contributed to the counterintuitive findings. Future studies should explore the reasons for such counterintuitive findings. We found that about 3% of the total participants reported having mental illnesses among family members. The lower percentage of mental illnesses might be related to the lower mental health literacy implying that despite using the vignettes participants did not recognize them as mental health conditions and attributed these to supernational entities or stigmatized beliefs. The results also suggested that about 96% of the total participants did not seek mental health care despite having mental illnesses at different courses of life. This is consistent with the National Mental Health Survey in Bangladesh [33] that revealed 92% of adults with mental health problems did not seek medical attention. Our results suggest this percentage is higher among indigenous people amid the shortage of mental health care and prevailing stigma in the CHT.

The socioeconomic factors considered risk factors for stigma toward mental illnesses may be closely linked to the social and cultural context of each indigenous community included in the study. Evidence suggests that social and cultural contexts determine the construct of stigma, endorsement of stigmatizing attitudes, and consequences [63, 64]. In addition, the experience of mental illnesses and potential causal factors precipitating the illnesses vary widely in relation to cultures and their diversities [65–67]. Therefore, the unique sociocultural context needs to be taken into consideration to understand the origin of stigma, meanings, and consequences [68]. Furthermore, examining indigenous concept, experience, and repercussions of mental illnesses may also be of paramount importance in order to address such illnesses in mental health care [25].

Regression analyses on seven subscales indicated association of different demographic variables. For example, number of family members and levels of income were found to be associated with treatability. Studies have shown the association between increased risk of mental illnesses and lower levels of income [69]. People with lower income may find it difficult to access mental health treatment. Treatability, that signifies a general belief that mental illnesses have proper treatment, therefore, can be related to lower income. It is unlikely to access mental health treatment unless the financial difficulty is resolved. However, higher levels of income have not always been shown to be strongly associated with lesser risk of mental illnesses [70, 71]. This suggests that treatability can be compounded with other factors besides income levels. Knowledge about mental health and access to mental health treatment among others may influence the belief of treatability of mental illnesses. Research highlighting the relationship between the number of family members and treatability especially among indigenous communities is scant. It is reasonable to assume that families having more members may struggle with defusing proper and equal attention to each member within the family. Lower levels of income may also further affect this diffusion contributing to the perpetuation of mental illnesses stigma.

Mental illnesses are generally perceived in terms of thoughts, emotions, and behaviors that eventually dictate the relationship with others [72]. Stigma attached to mental illnesses may, therefore, further disrupt social relationships. For example, some may think maintaining relationships and having faith in people with mental illnesses is difficult and risky. Evidence suggests that mental illnesses are associated with social distance [73] contributing to the stigmatized attitudes toward mental illnesses. Age, gender, and SES were found to be associated with relationship disruption. Age and gender were found to be associated with mental illnesses with evidence suggesting that stigma toward mental illnesses is less prevalent among younger persons and women [74]. Age, gender differences, and SES can determine the types and degree of stigmatized attitude toward mental illnesses. Research is needed to understand whether these variables (age, gender, and SES) are associated with similar relationship disturbances caused by mental illnesses stigma with regards to diverse indigenous communities.

Subscale hygiene was significantly associated with age, gender, SES, and knowledge about mental health. Evidence suggests both males and females reporting depression, has an increased likelihood of poor hygiene [75]. Therefore, people with mental illnesses may be stigmatized as incapable of maintaining personal hygiene. People struggling with maintaining hygiene due to mental illnesses can also be subjected to stigmatized attitudes and poor hygiene may be viewed as having a lack of mental health literacy and lower SES. Both qualitative and quantitative studies are required to understand the underlying mechanisms as to how age, gender, SES, and previous knowledge about mental health moderate mental illnesses stigma among indigenous communities.

Anxiety that governs mental illnesses stigma was found to be associated with age, gender, and SES. It is generally believed that anxiety increases with age [76], is more prevalent among women [77, 78], and is associated with SES [79]. However, little is known whether these variables underlie the anxiety of mental illnesses stigma, especially for indigenous people.

Professional efficiency, another subscale of the mental illnesses stigma, was associated with marital status, previous treatment history, and mental health literacy. People who have mental health literacy are likely to seek mental health treatment compared to those with little or no mental health literacy at all. In addition, people who seek treatment may believe that mental health professionals are capable of dealing with mental illnesses. Therefore, beliefs in the absence of professional efficiency may prevent people with mental illnesses from seeking treatment and contribute to the strengthening of mental illnesses stigma. However, the relationship between marital status and professional efficiency emerging from stigmatized attitudes is less studied. Evidence showed that marital status can influence the attitude toward mental illnesses [47]. Little research exists to study the relationship between marital status and professional efficiency associated with mental illnesses stigma for indigenous people around the world including in Bangladesh.

The present study is the first of its kind to investigate stigma toward mental illnesses among indigenous communities in Bangladesh. Results help understand the sociocultural factors associated with mental illnesses stigma among indigenous communities. The study offers further evidence that stigma toward mental illnesses is prevalent among indigenous communities in Bangladesh. The results may help inform the design of anti-stigma campaigns considering indigenous cultural and contextual factors.

Results should be interpreted in light of certain limitations. The cross-sectional design means directionality of the relationships cannot be confirmed. The limited sampling from one geographical location means that results cannot be generalized to all indigenous communities in Bangladesh. In addition, the overrepresentation of an indigenous community (i.e., Chakma- almost half of the total population) could overshadow the results leading to the restricted generalizability of the research findings. The Chakma community is the largest in the CHT, therefore, the overrepresentation might indicate a potential bias in sample selection. However, a recently conducted qualitative study found that mental illnesses stigma is prevalent among all indigenous communities in the CHT irrespective of size of the community [80]. Future studies should ensure the proportionate representation of all indigenous communities in the CHT to make precise generalization. Exploration of stigma among other indigenous communities in the CHT and living in other parts in Bangladesh (e. g., Northern parts) is recommended in future studies. The difference in reporting stigma toward mental illnesses in relation to gender should be cautiously interpreted. The disproportionate gender distribution in the sample should be considered in light of the population characteristics in Rangamati district and recruitment challenges due to remoteness. The distribution of male and female in Rangamati is about 54% and 46%, respectively [81]. Underrepresentation of women in the current study may be attributed to the skewed population distribution in Rangamati (the number of males is higher than females). In general, systemic barriers (e.g., gender inequality and discrimination), societal norms, gender roles, and cultural practices such as caregiving, domestic chores, or cultural expectations may create barriers eventually limiting women’s participation in research. In Bangladesh, women including those from indigenous communities have multiple roles and responsibilities including family, work, and community obligations making it difficult to allocate time for research activities leading to underrepresentation. Finally, the majority of people live in remote hill tracts areas in which accessing participants taking the equal gender distribution into account can be challenging. Future research can benefit with the fair representation of each gender.

We also acknowledge that response bias and purposive sampling techniques may restrict the generalization of the findings. It is possible that the study may have missed the inherent cultural factors (e.g., understanding of mental health) as independent factors among indigenous communities. The study could further be improved with qualitative exploration of the topic and investigation into how attitudes and stigma develop and sustain across the life-course in terms of the impact of treatment-seeking behavior.

Multiple comparisons may introduce potential limitations in the study findings with the chances of making Type 1 error. This increases the likelihood of observing significant differences by chance when multiple pairwise comparisons are performed leading to erroneous conclusions and false discoveries. Furthermore, multiple comparisons may subtly contribute to the reporting and highlighting of statistically significant findings disregarding non-significant findings. This may lead to biased reporting and eventually a skewed representation of overall findings. Future studies should use planned comparisons (e.g., predefined specific comparisons of interest based on theoretical hypotheses or contextual factors).

The translated version of the Day’s Mental Illness Stigma Scale was used in the study due to unavailability of scales or tools to assess mental illness stigma in Bangladesh. As previously mentioned, the translated scale was used by NIMH in association with the World Health Organization (WHO) in 2019 to understand the prevalence of mental illnesses in Bangladesh. Using translated version of a scale may raise concerns, especially regarding the robustness across cultures. However, the scale underwent adequate linguistic and cultural adaptation to ensure that the translated scale was appropriate and understandable to the target population. Use of the translated version seemed to be a practical option considering the lack of validated scale or measures in Bangla language. Nevertheless, we acknowledge the need for scales that are psychometrically sound and valid to avoid potential bias and inaccurate or misleading findings.

Finally, several opportunities to assess cultural factors that may impact stigma have not been adequately addressed within the limited scope of the study. For example, cultural norms and values of a given geographical location with distinct languages and communication styles may affect data collection methods and response rate. Therefore, it is crucial to understand cultural norms when collecting data to ensure its reliability and accuracy. Furthermore, power dynamics, social inequalities, hierarchical structures, cultural biases as well as stereotypes, and cultural attitudes toward data collection and research within a society may affect the willingness of individuals to provide accurate responses. Understanding the social context is crucial to account for these factors. These factors should be taken into consideration in future research conducted in this context.

## Conclusions

Mental illnesses stigma is present among indigenous communities in Bangladesh. Age, gender, socioeconomic status, and income were associated with stigma toward mental illnesses among indigenous communities in the Chattogram Hill Tracts. The findings may have implications for government and non-government policy makers and stakeholders in designing targeted mental health strategies and interventions to reduce stigma and increase mental health literacy in this region.

## Data Availability

The data and materials can be shared with the approval of the Ethical Review Committee at the Department of Clinical Psychology, University of Dhaka, Bangladesh. The request can be made to the corresponding author.
